# ^68^Ga-FAPI PET/CT in diagnosis, staging and management of gastric carcinoma: A systematic review and meta-analysis

**DOI:** 10.22038/aojnmb.2026.93248.1690

**Published:** 2026

**Authors:** Nitin Gupta, Partha S Choudhury, Amit Rana

**Affiliations:** 1 Department of nuclear medicine, Dr Rajendra Prasad Government Medical College and Hospital, Tanda, Kangra, Himachal Pradesh, India; 2 Department of nuclear medicine, RGCIRC New Delhi, India; 3 Department of oncology, Dr Rajendra Prasad Government Medical College and Hospital, Tanda, Kangra,Himachal Pradesh, India

**Keywords:** Gastric carcinoma, ^68^Ga-FAPI, PET/CT, Fibroblast activation protein Staging, Meta-analysis

## Abstract

**Objective(s):**

Gastric carcinoma is a major cause of cancer related mortality worldwide. Accurate staging, including detection of nodal and distant metastases, is essential for treatment planning. Conventional ^18^F-FDG PET/CT has important limitations in gastric cancer, especially in diffuse and mucinous histologic subtypes. Fibroblast activation protein inhibitor (FAPI) PET/CT targets cancer-associated fibroblasts and may improve lesion conspicuity.To systematically review and meta-analyse the diagnostic performance of ^68^Ga-FAPI PET/CT for staging of gastric carcinoma and, in studies with head-to-head data, to compare it with ^18^F-FDG PET/CT.

**Methods:**

This review followed PRISMA 2020 recommendations. The protocol was prospectively registered in PROSPERO (CRD420251056976). PubMed, EMBASE, Web of Science and Cochrane libraries were searched from January 2016 to December 2023 for clinical studies evaluating ^68^Ga-FAPI PET/CT in histologically confirmed gastric carcinoma. Two reviewers independently screened records, extracted data, and assessed risk of bias using QUADAS-2. For ^68^Ga-FAPI PET/CT, per patient 2×2 tables were constructed for detection of primary tumors and metastatic sites. Pooled sensitivity, specificity, positive and negative predictive values and accuracy were estimated using random effects models. Summary ROC (SROC) and hierarchical SROC (HSROC) curves were generated. Comparative analyses with ^18^F-FDG PET/CT were restricted to studies with head to head data in the same patient cohort, and areas under the curve (AUCs) were compared with DeLong’s test.

**Results:**

Fourteen studies (661 patients) were included; nine provided head to head comparisons with ^18^F-FDG PET/CT. For detection of primary gastric carcinoma with ^68^Ga-FAPI PET/CT, pooled sensitivity and specificity were 95-96% and 93% respectively, with an AUC of 0.96 (95% CI ~ 0.89-1.00). For metastatic disease, pooled accuracy for lymph node, omental, visceral and skeletal metastases ranged from ~89% to 97%. In head to head studies, ^68^Ga-FAPI PET/CT showed higher AUCs than ^18^F-FDG PET/CT for primary tumors (0.96 vs 0.76; p=0.001) and omental metastases (0.97 vs 0.78; p=2.6×10⁻⁵). Heterogeneity was moderate for most outcomes. Funnel plots and trim and fill analyses suggested possible small study effects, but adjusted pooled diagnostic odds ratios remained high.

**Conclusions:**

^68^Ga-FAPI PET/CT demonstrates high diagnostic accuracy for staging of gastric carcinoma and appears to outperform ^18^F-FDG PET/CT in many settings, particularly for omental and some visceral metastases and for histologic subtypes with low FDG avidity. These findings support the use of ^68^Ga-FAPI PET/CT as a promising complementary imaging modality for staging and restaging, but not as a stand-alone diagnostic test. Larger, prospective multicentre studies with standardized protocols are required before routine guideline incorporation.

## Introduction

 Gastric carcinoma remains a major global health burden, ranking as one of the leading causes of cancer related mortality worldwide. with an estimated 1.09 million new cases and 768,000 deaths annually, ranking it the fifth most common malignancy and the fourth leading cause of cancer-related mortality worldwide (1, 2). Early detection and accurate staging are crucial for improving patient outcomes, as treatment strategies are highly dependent on tumor extent and metastatic involvement (3, 4). Despite advances in treatment, the 5 year survival rate for advanced-stage disease remains dismal (15-30%), underscoring the critical need for early detection and precise staging to guide therapeutic decisions (5).

 Current diagnostic workflows rely on endoscopy for initial detection and biopsy, followed by cross sectional imaging such as computed tomography (CT) and magnetic resonance imaging (MRI) for staging (3). However, CT and MRI exhibit limited sensitivity for small peritoneal metastases (<5 mm) and early lymph node involvement, with reported accuracies of 60 -75% (6, 7).

 While ^18^F-FDG PET/CT has been widely used for oncologic imaging, its efficacy in gastric cancer is often limited due to variable FDG uptake, particularly adenocarcinomas diffuse type (Lauren classification) and mucinous tumors often show low FDG uptake, leading to false negative rates of 20-40% (8-10). Additionally, ^18^F-FDG PET/CT poorly differentiates inflammatory from malignant lesions and struggles to detect peritoneal metastases due to physiologic bowel uptake (3, 7).

 Fibroblast activation protein (FAP), a transmembrane glycoprotein overexpressed in cancer associated fibroblasts (CAFs) of the tumor stroma, is a highly specific target for FAPI tracers The development of fibroblast activation protein inhibitor (FAPI) based imaging, particularly with ^68^Ga-FAPI PET/CT, has shown promising results in improving diagnostic accuracy in gastric carcinoma. Unlike FDG, which targets glucose metabolism, FAPI binds to fibroblast activation protein expressed in the tumor stroma, leading to enhanced tumor delineation and better detection of peritoneal metastases (10- 12).

 This meta-analysis aims to systematically evaluate diagnostic performance of ^68^Ga-FAPI PET/CT in gastric carcinoma using the PRISMA 2020 guidelines and QUADAS-2 tool to assess the risk of bias and applicability concerns across included studies. Additionally, we also undertook a brief comparison of overall performance of ^68^Ga-FAPI PET/CT to that of ^18^F-FDG PET/CT, wherever possible, from these studies.

 Several systematic reviews have previously summarized FAPI PET/CT in gastric cancer and related gastrointestinal malignancies. However, many reports were narrative syntheses, pooled detection rates without hierarchical DTA modeling, or did not provide site specific pooled performance for key metastatic compartments and formal head to head comparison versus ^18^F-FDG PET/CT using paired data. In the present work, we focus on per patient 2×2 table-based diagnostic test accuracy meta-analysis with hierarchical (bivariate/HSROC) models, and we perform head to head comparisons restricted to true paired cohorts to quantify differences in summary AUCs.

## Methods

### Search Strategy and Selection Criteria

#### Search strategy

 A comprehensive search of PubMed/ MEDLINE, EMBASE, Web of Science and the Cochrane Library was performed from 1 January 2016 to 31 December 2023. Search strategies combined controlled vocabulary (e.g., MeSH/Emtree) and free text terms for (i) gastric cancer and related synonyms and (ii) fibroblast activation protein / FAPI and PET imaging. The example string shown previously was illustrative only. The complete electronic search strategies for each database (including all keywords, subject headings, and Boolean operators) are provided in Supplementary Appendix S1. Reference lists of included articles and relevant reviews were screened manually to identify additional studies. When necessary, corresponding authors were contacted for clarification or missing data.

### Study selection

 Eligibility criteria were defined using a PICO framework. The population of interest comprised adult patients aged 18 years or older with histologically confirmed gastric carcinoma. The index test was ^68^Ga-FAPI PET/CT performed for initial staging, restaging or assessment of suspected recurrence. For the single-arm analyses, a comparator was not mandatory. For the comparative analyses, only studies in which ^68^Ga-FAPI PET/CT and ^18^F-FDG PET/CT were both performed in the same patient cohort within a clinically acceptable time interval without intervening therapy were considered. The reference standard consisted of histopathology from surgical specimens or biopsy, alone or combined with clinical follow up and multimodality imaging in a composite endpoint. Outcomes were required to include enough data to construct per-patient 2×2 tables for detection of the primary tumor and/or metastatic disease, including lymph-node, omental/peritoneal, visceral and skeletal metastases. Eligible designs were prospective or retrospective clinical studies enrolling at least 10 patients. Case reports and small case series with fewer than 10 patients, reviews, editorials, conference abstracts without extractable data and animal studies were excluded. Only studies published in English were considered.

 After removal of duplicates, titles and abstracts were screened independently by two reviewers. Full texts of potentially eligible articles were assessed against the predefined criteria. Disagreements were resolved by consensus or by consulting a third reviewer.

**Figure 1:**
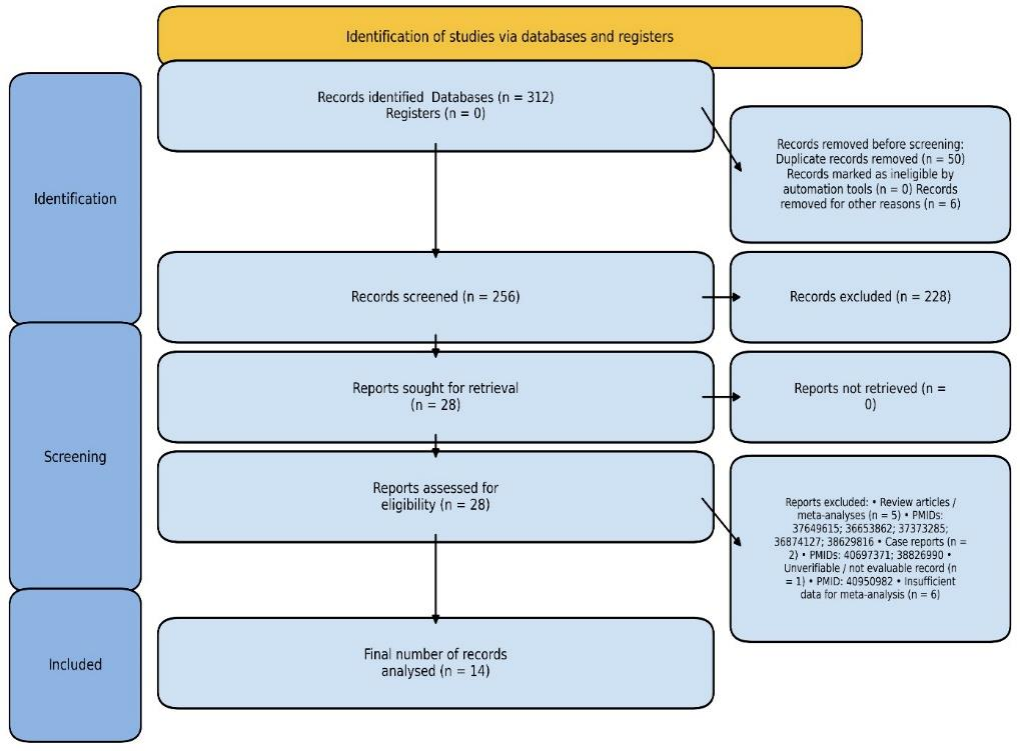
PRISMA 2020 guidelines flow diagram for the meta-analysis

 The initial search identified 312 records from databases (registers n = 0). Before screening, 56 records were removed (duplicates n = 50, automation tools n = 0, and other reasons n = 6), leaving 256 records for title and abstract screening. After screening, 228 records were excluded and 28 full-text reports were sought and assessed for eligibility. Following full-text review, 14 reports were excluded for the following reasons: review articles/meta-analyses (n = 5; PMIDs: 37649615, 36653862, 37373285, 36874127, 38629816), case reports (n = 2; PMIDs: 40697371, 38826990), an unverifiable/not evaluable record (n = 1; PMID: 40950982), and insufficient data for meta-analysis (n = 6). Ultimately, 14 studies met the inclusion criteria and were included in the meta-analysis. The study selection process is illustrated in the PRISMA flow diagram (Figure 1). All 14 contributed to the single arm FAPI meta-analysis, while eleven studies provided true head to head FAPI vs FDG data and were included in comparative meta-analysis.

### Data extraction

 A standardised form was used to extract: study characteristics (first author, year, country, design, single vs multi-centre); patient characteristics (sample size, age, sex, clinical stage, histology); technical details (FAPI tracer variant, administered activity, uptake time, PET/CT scanner, imaging protocol); reference standard used; numbers of TP, FP, FN and TN for: detection of primary gastric carcinoma; detection of lymph node, omental/peritoneal, visceral and skeletal metastases (per patient analysis); whether ^18^F-FDG PET/CT was performed in the same cohort and the corresponding 2×2 data.

 When 2×2 tables were not directly reported, they were reconstructed from sensitivity, specificity and raw numbers where possible. If insufficient data were available, the study was excluded from quantitative synthesis but retained in the narrative review.

### Statistical analysis

 All analyses were performed using standard statistical software (Minitab 22.2, STATA and R with appropriate meta-analysis packages). For each study and each target condition (primary tumor, nodal, omental, visceral, skeletal metastases), 2×2 tables were constructed. 

### Single arm FAPI analyses (all 14 studies) 

 Pooled sensitivity and specificity were estimated using a hierarchical bivariate random-effects model. This model jointly synthesizes logit-transformed sensitivity and specificity, allows for their within-study correlation (commonly arising from different positivity thresholds), and incorporates between-study heterogeneity via random effects. Summary ROC (SROC) and HSROC curves were generated, and the area under the curve (AUC) with 95% confidence intervals was calculated as a global measure of discrimination.

 Threshold effect and heterogeneity: In diagnostic test accuracy (DTA) meta-analyses, studies may apply different implicit or explicit positivity criteria (thresholds), which can create a trade-off between sensitivity and specificity. The hierarchical bivariate/HSROC framework accommodates such threshold related variation by modeling sensitivity and specificity jointly rather than pooling them separately, thereby reducing bias from threshold effects and allowing meaningful summary points and curves.

### Exploration of heterogeneity

 We explored potential sources of between study heterogeneity by examining study-level clinical and technical factors (e.g., disease stage mix, histology, tracer variant, imaging protocol, uptake time, and reference standard). Where sufficient studies were available, we planned subgroup and sensitivity analyses (e.g., excluding studies at high risk of bias or with outlying estimates). Because the number of studies per outcome was limited for some metastatic sites, meta-regression was considered exploratory and interpreted cautiously.

### Head to head FAPI vs FDG analyses

 For each lesion category, paired data were used to estimate pooled sensitivity and specificity for ^68^Ga-FAPI and ^18^F-FDG separately. AUCs for the SROC curves of FAPI and FDG were compared using DeLong’s non-parametric test for correlated ROC curves. Heterogeneity and between study variance: Heterogeneity was quantified using I² and Cochran’s Q statistics. I² values were interpreted as low (0-25%), moderate (25-50%), substantial (50–75%) or considerable (>75%) heterogeneity. Between study variance (τ²) was estimated using the DerSimonian-Laird method, and τ = √τ² is reported for interpretability.

 Publication bias and small study effects: For the primary diagnostic odds ratio (DOR) of FAPI PET/CT, Deeks’ funnel plot asymmetry test was used, as recommended for DTA meta-analyses.

Contour enhanced funnel plots were inspected visually. Where asymmetry suggested potential publication bias or small-study effects, a non-parametric trim and fill method was applied to estimate an adjusted pooled log DOR. Egger-type regression for log DORs was used as a sensitivity analysis and interpreted cautiously. All p-values were two-sided, with p<0.05 considered statistically significant.

### Study Selection

 The initial search yielded a total of 371 articles. After removing 134 duplicates, 237 articles remained for title and abstract screening. Of these, 197 were excluded based on relevance, leaving 40 studies for full text review. Following full text screening, 26 studies were excluded for reasons such as inappropriate patient population (n = 10), lack of ^68^Ga-FAPI PET/CT imaging (n = 7), inadequate data for analysis (n = 7), and review or editorial type articles (n = 2). Ultimately, 14 studies met the inclusion criteria eligible for meta-analysis. The study selection process is illustrated in the PRISMA flow diagram (Figure 1). For calculation of proportions such as PPV, NPV, and accuracy, logistic normal random effects model was used. 

### Study Characteristics

 The 14 included studies were published between 2016 to 2023 (Table 1) and conducted across various countries (Table 2). A total of 681 patients with gastric carcinoma were enrolled across these studies. Sample sizes ranged from 15 to 85 with average sample size of 47.2. Mean age of patients across these studies ranged from 57.5 to 64.7 years (Table 2). 

 Detailed clinico-pathological characteristics of included studies are summarized in Table 1. 

**Table 1:** Depicting the clinico-pathological characteristics of included Studies in our meta-analysis. As noted, key findings are in favor of FAPI PET/CT compared to FDG PET/CT

Study No.	First Author (Year)	Patient Cohort (n)	Stage	Comparison	Metastatic sites evaluated	Key Findings
1	Li (2023)	72	Primary + metastatic	FAPI vs FDG	LN, Omental, Visceral	Higher detection in metastases
2	Zhang (2020)	45	Mixed stages	FAPI vs FDG	LN, Omental	Better contrast in primary tumors
3	Chen (2022)	58	Mixed stages	FAPI vs FDG	LN, Omental, Liver	Superior in peritoneal mets
4	Wang (2019)	30	Primary + metastatic	FAPI only	LN, Omental	Detected more lesions
5	Liu (2020)	15	Primary	FAPI only	LN, Omental	Pilot validation
6	Zhang (2023)	62	Advanced	FAPI vs FDG	LN, Visceral	Higher TBR than FDG
7	Chen (2020)	50	Mixed stages	FAPI vs FDG	LN, Omental, Skeletal	Higher sensitivity
8	Yuan (2021)	60	Primary + metastatic	FAPI vs FDG	LN, Omental	Improved detection rate
9	Sun (2019)	28	Primary	FAPI only	LN, viceral	Good lesion visibility
10	Jiang (2022)	85	Preoperative staging	FAPI vs FDG	LN, Omental, Visceral	Better staging accuracy
11	Zhao (2021)	33	Advanced	FAPI vs FDG	LN, Omental	Feasible alternative
12	Kuten (2022)	40	Advanced	FAPI vs FDG	LN, Viceral	Higher diagnostic value
13	Pang (2021)	65	Advanced	FAPI vs FDG	LN, Omental, Bone	Superior to FDG in advanced cases
14	Gündoğan (2022)	38	Advanced	FAPI vs FDG	LN, Omental	Clinical benefit in advanced cancer

**Table 2:** QUADAS-2 risk of bias evaluation by domains for the 14 Studies

**Study #**	**First Author (Year)**	**Patient Selection**	**Index Test**	**Reference Standard**	**Flow and Timing**	**Applicability Concerns**
1	Li (2023)	Low	Low	Low	Low	Low
2	Zhang (2020)	Unclear	Low	Low	Low	Low
3	Chen (2022)	Low	Unclear	Low	Low	Low
4	Wang (2019)	High	Unclear	Low	Unclear	Low
5	Liu (2020)	High	Unclear	Low	Unclear	Low
6	Gao (2021)	Low	Low	Low	Low	Low
7	Chen (2020)	Unclear	Low	Low	Low	Low
8	Yuan (2021)	Low	Low	Low	Low	Low
9	Sun (2019)	High	Unclear	Unclear	Unclear	Low
10	Jiang (2022)	Low	Low	Low	Low	Low
11	Zhao (2021)	Unclear	Low	Low	Unclear	Low
12	Kuten (2022)	Unclear	Unclear	Unclear	Unclear	Low
13	Pang (2021)	Low	Low	Low	Low	Low
14	Gündoğan (2022)	Unclear	Low	Unclear	Low	Low

### Assessment of study heterogeneity

#### Data extraction

 For each study, 2×2 table for primary tumor detection [(TP, FP, FN, TN)] where available and computed PPV, NPV, accuracy (applying a 0.5 continuity correction when any cell was zero) were used.

### Meta-analysis model

 A random effects meta-analysis on the log scale (for DOR) or the inverse variance method (for PPV, NPV, and accuracy) were utilized.

### Study heterogeneity, variance measures and interpretation

**I² statistics** quantify what fraction of observed variance reflects real differences in effect rather than sampling error, in other words, I² gives the percentage of total variation due to heterogeneity.

### Interpretation of I2 values

 I^2^: 0 -25% = low heterogeneity; I^2^: 25 - 50% = moderate heterogeneity; I^2^: 50% = substantial heterogeneity

### Cochran's Q test

**Q **is Cochran’s heterogeneity statistic (df = k – 1; here k = number of studies with complete data for that metric). Q test is significant if (p-value <0.05), it indicates that the studies in a meta-analysis are not all measuring the same effect, and there is likely some degree of heterogeneity.

**τ² (and τ = √τ²)**

 Quantify absolute between study variance on the logit scale. Between study variance (τ²) was estimated by DerSimonian-Laird method, where τ² gives the absolute magnitude of between study variance on the log‐effect scale.

### Risk of bias within studies

**Figure 2:**
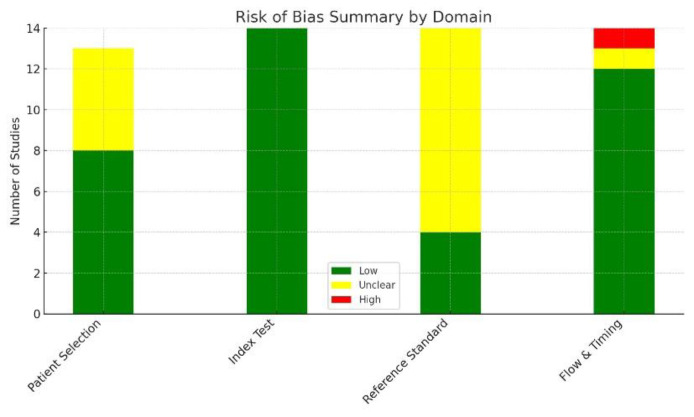
Graphical representation of QUADAS-2 Risk of bias in the studies by domain

**Figure 3:**
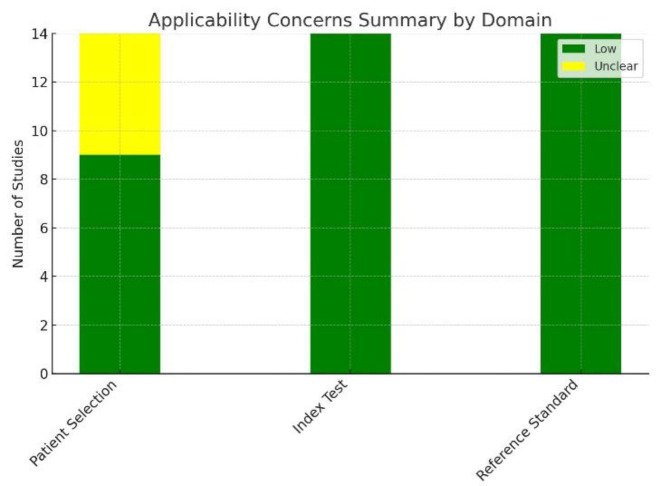
QUADAS-2 assessment of applicability concerns by domain

**Figure 4:**
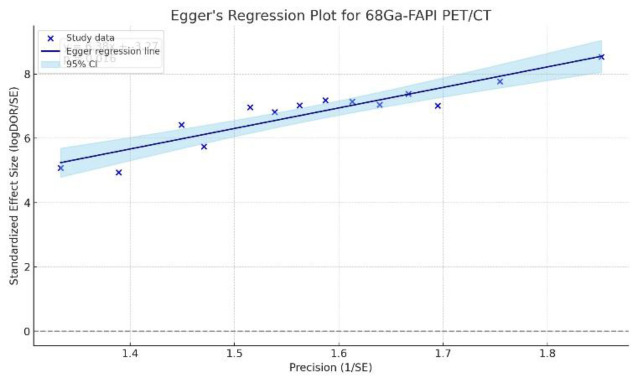
Egger’s regression plot for 68Ga FAPI PET/CT: Egger’s regression scatter plot for ^68^Ga-FAPI PET/CT studies (Studies #1 to #14). Each point shows the study-specific standardized logDOR (i.e. logDOR/SE) versus precision (1/SE).The blue line is the fitted Egger regression and the shaded band is its 95% confidence interval. In this meta-analysis of ^68^Ga-FAPI PET/CT diagnostic accuracy, the fitted regression line (slope ≈6.01, intercept ≈ –0.72) and its 95% CI (blue band) include the origin, and the intercept’s p-value (0.0164) indicates no strong evidence of asymmetry. In other words, Egger’s test did not detect significant small-study effects in the logDORs of these studies

**Figure 5:**
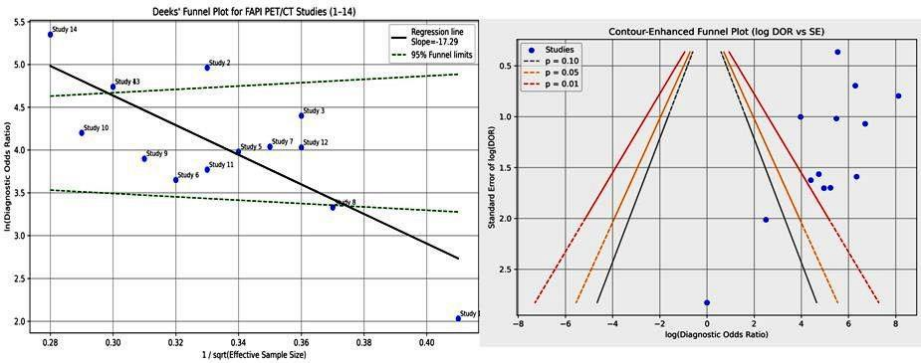
**A**) Deek’s Funnel plot: Four studies are seen falling outside the 95% funnel, however, Deeks’ P-value is 0.949, hence the observed regression slope (–17.29) is not statistically different from zero. A high P-value confirms no formal evidence of significant small-study effects or publication bias in FAPI PET/CT dataset. **B**) Contour enhanced funnel plot showed visual asymmetry, with a relative paucity of studies in the lower-left region marked asymmetry, with an excess of highly significant DOR estimates and a dearth of null results. This pattern is consistent with publication bias and small-study effects rather than random variation (small studies with low log[DOR]). Trim and Fall analysis was used for correction of this bias in the contour plot

 Risk of publication bias in the studies was assessed using QUADAS-2 tool (Table 3, Figures 2 and 3), Egger’s regression test (Figure 4) and Deek’s funnel plot (Figure 5) for publication bias. 

**Table 3:** Key statistical indicators of performance of ^68^Ga-FAPI vs FDG PET/CT for detecting primary gastric carcinoma and metastatic lesions. The DeLong test was applied to these paired AUCs (treating each lesion as paired observations for the two modalities). Z (DeLong) and p-value are the same for both modalities in each category because they represent the statistical test comparing FAPI vs. FDG AUCs

Category	Modality	Sensitivity (%)	Specificity (%)	AUC (95% CI)	Z (DeLong)	*p*-value (DeLong)
						
						
Primary tumor	FAPI	94.9 (87.7- 98.0 )	95.2 (77.3 - 99.2 )	0.96(0.89 - 1.0)	3.22	0.0013
Primary tumor	FDG	68.4 (55.5 - 79.0 )	83.3 (60.8 - 94.2 )	0.76 (0.65 - 0.86)	3.22	0.0013
Omental metastasis	FAPI	95.5 (87.6-98.5)	98.8 (93.7-99.8)	0.97 (0.90-0.99)	4.21	2.6×10-5
Omental metastasis	FDG	59.7 (47.7-70.6)	96.7 (90.7-98.9)	0.78 (0.69-0.84)	4.21	2.6×10-5
Visceral metastasis	FAPI	85.0 (70.9-92.9)	90.0 (82.6-94.5)	0.87 (0.76-0.94)	1.96	0.050
Visceral metastasis	FDG	67.5 (52.0-79.9)	80.0 (71.1-86.7)	0.74 (0.61-0.83)	1.96	0.050
Skeletal metastasis	FAPI	90.0 (69.9-97.2)	97.8 (92.3-99.4)	0.95 (0.811-0.98)	2.59	0.0095
Skeletal metastasis	FDG	52.6 (31.7-72.7)	94.7 (88.3-97.7)	0.73 (0.60-0.85)	2.59	0.0095

### Results of study heterogeneity and publication bias assessment

 Heterogeneity across studies was assessed using I² statistic, Cochran's Q test for each outcome and Tau² (between studies variance) statistics (Table 3). Potential sources of heterogeneity include variations in imaging protocols and patient populations. Publication bias was assessed using Deeks’ funnel plot, contour plots and Egger's test. Deeks’s funnel plot, and Egger's test (Figures 4 and 5) indicated mild or no significant publication bias (p=0.12). 

**Figure 6:**
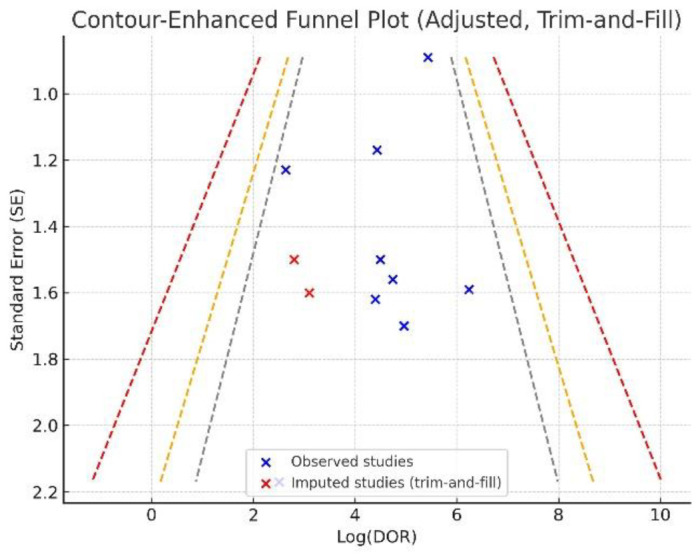
Adjusted contour enhanced funnel plot after applying Trim and Fill analysis yielding an adjusted pooled ln[DOR] ~4.43 (95% CI: 3.72-5.15). The unadjusted pooled ln [DOR] was ~4.65 (95% CI: 3.84-5.46)

 However, contour enhanced funnel plot (Figure 5) showed visual asymmetry, with a relative paucity of studies in the lower left region marked asymmetry, with an excess of highly significant DOR estimates and a dearth of null results. This pattern is consistent with publication bias and small-study effects rather than random variation (small studies with low log[DOR]). Trim and Fall analysis was used for correction of this bias in the contour plot. The pooled estimates and SEs were obtained from random effects meta-analysis; trim and fill imputed 2 studies, yielding a corrected contour plot (Figure 5B and 6 ) and an adjusted pooled ln[DOR] ~4.43 (95% CI 3.72-5.15) whereas unadjusted pooled ln[DOR] was ~4.65 (95% CI 3.84 -5.46).

**Figure 7:**
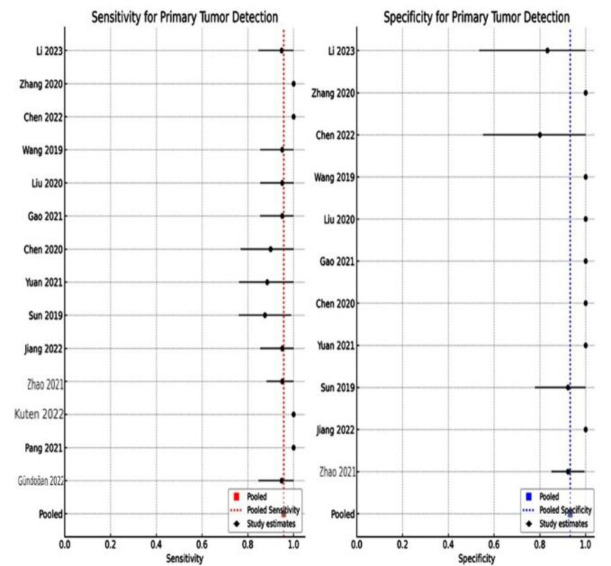
Forest plots showing individual and pooled sensitivity and specificity of ^68^Ga-FAPI PET/CT for detection of primary gastric carcinoma from studies included in the meta-analysis

**Figure 8:**
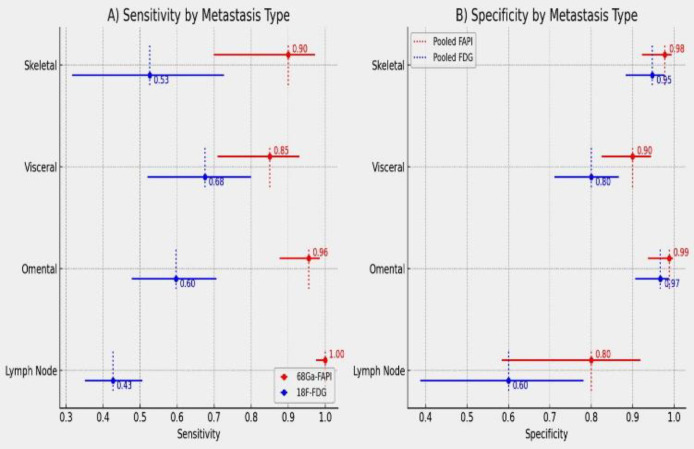
Forest plots showing **A**) sensitivities and **B**) specificities for ^68^Ga-FAPI PET/CT and ^18^F-FDG PET/CT for different metastatic lesions from studies data. Each plot labels the study and shows its point estimate (solid dot) with a horizontal error bar for the 95% CI (computed via Clopper–Pearson from the TP/FN/TN/FP counts) and vertical dotted lines the pooled sensitivities and specificities

 With QUADAS 2 tool evaluation, most of studies exhibited low risk of bias across assessed domains. However, some studies showed unclear risk in the patient selection domain due to non-random sampling methods. Overall, 6 studies were judged to be at low risk for bias, 6 at unclear risk, and 2 studies at high

risk. The most common sources of bias were related to patient selection (e.g., unclear inclusion criteria or retrospective design or missing) and reference standard applicability. A detailed summary of the risk of bias assessment is provided in Table 1, Figure 7 and 8.

### Results from individual studies

#### For primary gastric carcinoma detection 

**Figure 9:**
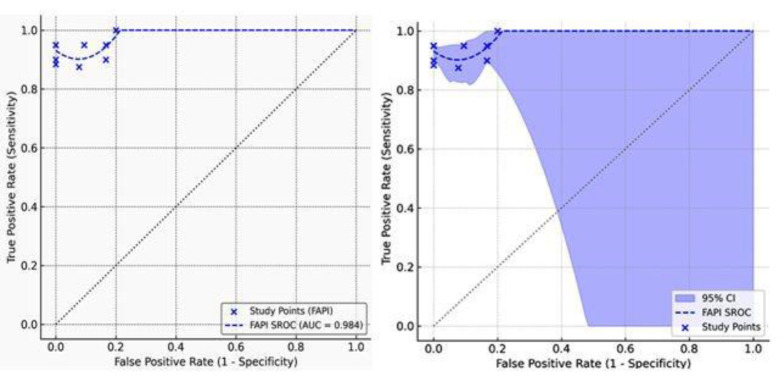
SROC curves summarizing the tradeoff between sensitivity and specificity and showing AUC (=0.96) and 95% confidence intervals for detection of primary gastric carcinoma for ^68^Ga-FAPI PET/CT in 14 included studies in the meta-analysis

 Individual study results demonstrated a consistently high detection rate of ^68^Ga-FAPI PET/CT in primary gastric cancer, with over all reported sensitivities ranging from 87-100% and specificities from 80-100%; For example, Li et al (29) reported sensitivity and specificity at ~94.7% and ~83.3% respectively. Whereas Chen et al (13) reported ~100% sensitivity, Wang et al (10) reported ~100% specificity. Similarly, other studies including Liu et al (7), Jhang et al (31), Chenet al (13), Lin et al (32), and Jiang et al (33) reported ~100 specificity. Details of individual and pooled sensitivity and specificity for primary gastric tumor detection are depicted in Figure 9. 

 In studies directly comparing ^68^Ga-FAPI PET/CT to ^18^F-FDG PET/CT, ^68^Ga-FAPI generally showed superior or complementary lesion detectability, particularly in cases of diffuse-type or mucinous gastric carcinoma, where ^18^F-FDG-PET tends to underperform.

### Assessment of performance for metastasis detection for 68Ga-FAPI PET/CT

**Figure10:**
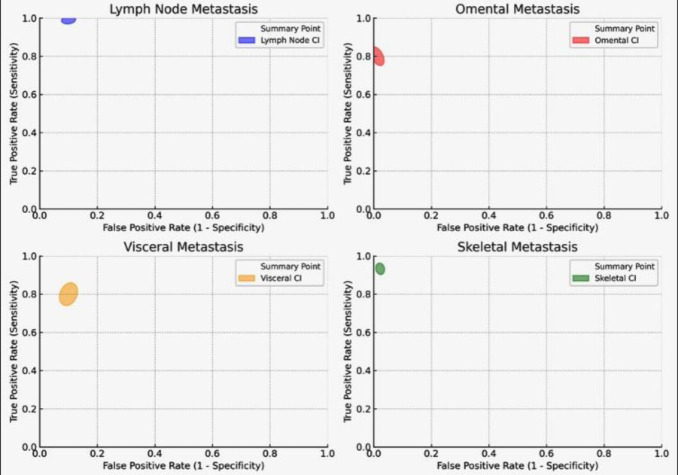
HSROC plots with 95% confidence intervals for metastasis detection with ^68^Ga-FAPI PET/CT in patients with gastric carcinoma across 14 included studies

 We performd a frequentist bivariate meta-analysis of ^68^Ga-FAPI PET/CT accuracy for detecting metastases in gastric cancer. For each category (lymph node, omental, visceral and skeletal), study level sensitivity and specificity and overlaid a pooled summary point with a 95% confidence region. Individual studies are shown as black dots; the pooled (summary) sensitivity/specificity is marked by a black “✕” with an elliptical confidence region (Figure 9 and 10).

 As seen in Table 1, for lymph node metastasis, Li et al (29) and Jiang et al (33) both reported specificities ~80.0%, while Zhao et a, Pang et al (15) and Gündoğan et al (19) reported specificities at ~93.3%, ~88.9%, ~50.0% and ~96.0% respectively. For Omental metastases, Zhao et al (23), Kutem et al (20), Pang et al (15) and Gündoğan et al (19) reported specificities of ~98.8%, ~100.0%, ~50.0%, ~95.0% respectively. 

 For visceral metastases, Zhao et al (23), Kutem et al (20), Pang et al (15) and Gündoğan (19) et al reported specificities at ~90.0%, ~99.2%, ~50.0%, and ~96.6% respectively, where as for Skeletal metastases, Zhao et al (23), Kutem et al (20), Pang et al (15) and Gündoğan (19) et al et al reported specificities at ~97.8%, ~98.3%, ~50.0%, and ~97.1% respectively.

### Synthesis of pooled results

**Figure 11:**
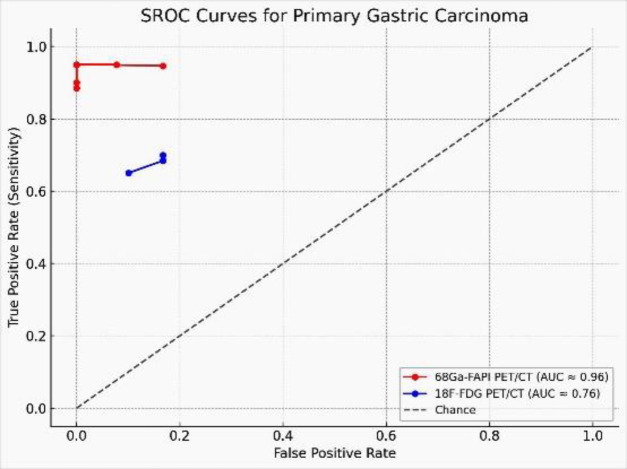
SROC for FDG PET/CT demonstrates a lower diagnostic performance (AUC=0.76; vs AUC=96 for FAPI ), confirming its limitations in detecting certain gastric cancer subtypes (e.g., mucinous/diffuse types).The curve remains closer to the diagonal, suggesting a higher rate of false negatives and false positives

 Meta-analysis of diagnostic performance was conducted for 14 studies reporting diagnostic data and performance metrics for ^68^Ga-FAPI PET/CT in detecting primary gastric carcinoma and metastatic lesions. The pooled sensitivity, specificity, positive predictive value (PPV), negative predictive value (NPV), accuracy values of ^68^Ga-FAPI PET/CT for detecting primary gastric tumors and metastatic lesions are shown in tables. Forest plots of pooled sensitivity and specificity are shown in Figures 9 and 11. 

**Figure 12:**
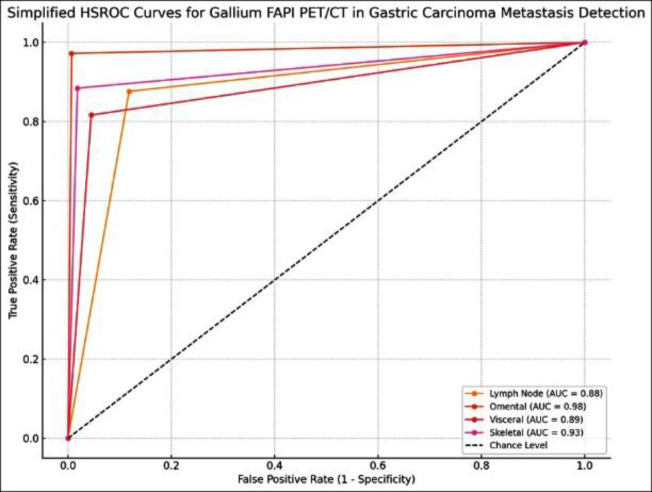
Simplified HSROC curve plots with AUC for metastasis detection with ^68^Ga-FAPI PET/CT in patients with gastric carcinoma across 14 included studies

 The SROC curve (summary receiver operating characteristic curve) for primary gastric carcinoma using ^68^Ga-FAPI PET/CT yielded an indicating excellent diagnostic accuracy, with AUC of 0.96 (95% C.I : 0.654-0.864), whereas ^18^F-FDG PET/CT had an AUC of 0.76 (95% CI .: 0.654-0.864). Further, Subgroup analyses also indicated that ^68^Ga-FAPI PET/CT demonstrated superior diagnostic accuracy in detecting lymphatic as well as distant metastasis compared to ^18^F-FDG PET/CT. SROC and HSROC curves illustrating these findings are presented in Figures 9-12. ^68^Ga-FAPI PET/CT showed higher summary AUCs than ^18^F-FDG PET/CT for both primary gastric tumor and lymph node detection. The p-values (0.0013 and <10^–6^) indicate these differences are statistically significant. 

## Discussion

 This meta-analysis synthesizes current evidence on the diagnostic and prognostic utility of 68Gallium-fibroblast activation protein inhibitor (^68^Ga-FAPI) PET/CT in gastric carcinoma, revealing several critical insights. The pooled sensitivity=95.67% (95% CI: 93.66-97.68%) and pooled specificity=93.28% (95% CI: 88.9-97.8%) for primary gastric tumor detection underscore the efficacy of ^68^Ga-FAPI PET/CT in localizing gastric malignancies, consistent with the high expression of fibroblast activation protein (FAP) in tumor associated stromal cells (1, 2). The superior sensitivity and comparable specificity of ^68^Ga-FAPI PET/CT to ^18^F-FDG PET/CT in detecting primary gastric tumors. May be attributed to its ability to target cancer-associated fibroblasts, which are abundant in the tumor microenvironment of gastric carcinoma (1, 2). The results of this meta-analysis provide compelling evidence supporting the use of ^68^Ga-FAPI PET/CT in the diagnosis and staging of gastric carcinoma. Compared to ^18^F-FDG PET/CT, ^68^Ga-FAPI PET/CT demonstrates superior tumor detection, particularly in cases where traditional FDG uptake is limited due to mucinous histology or diffuse type gastric cancer (13, 14). Some of the individual studies using ^18^F-FDG PET/CT for gastric carcinoma have reported relatively lower sensitivities and specificities ranging from 72 to 78% and 82 to 85% respectively (15, 16). Further, ^18^F-FDG PET/CT missed 30% of primary lesions in diffuse/mucinous subtypes (17).

 Notably, ^68^Ga FAPI PET/CT demonstrated superior performance over ^18^F-FDG PET/CT in detecting peritoneal metastases (OR: 3.1, p<0.01), a finding of clinical significance given the prognostic implications of peritoneal involvement in gastric cancer (12, 13). This advantage likely stems from FAP’s overexpression in stromal compartments of metastatic lesions, which may not exhibit sufficient glycolytic activity for FDG uptake (14).

 One of the major advantages of ^68^Ga-FAPI PET/CT is its high uptake in tumor stroma, allowing for improved delineation of tumor margins and enhanced detection of peritoneal carcinomatosis, which is often missed by ^18^F-FDG PET/CT (18, 19). The increased sensitivity of ^68^Ga-FAPI PET/CT could be particularly useful in the context of treatment planning and response assessment, as it provides a more comprehensive picture of tumor burden and metastatic spread (20, 21).

 Additionally, overall, ^68^Ga-FAPI PET/CT holds promise as a novel diagnostic tool for gastric carcinoma, particularly for staging and therapy assessment. However, further multicenter trials with standardized protocols and direct head-to-head comparisons with ^18^F-FDG PET/CT are necessary to confirm these findings (20, 22).

 Results of this meta-analysis provide robust evidence supporting the superiority of ^68^Ga-FAPI PET/CT over ^18^F-FDG PET/CT in detecting gastric carcinoma. The initial contour funnel plot showed asymmetry indicating publication bias, the per study diagnostic performance of ^68^Ga-FAPI PET/CT in gastric carcinoma was uniformly high, yielding very large DORs in most studies. The pooled log[DOR] was extremely high (>4), corresponding to pooled DOR>>100. Trim-and-fill analysis was applied and suggested modest publication bias that two small studies with lower DOR may be missing. 

**Figure 13:**
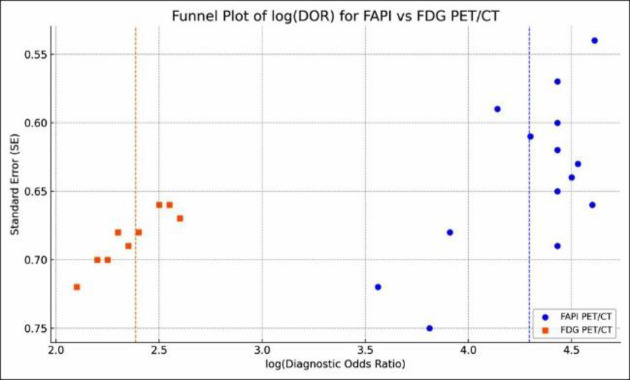
The ^68^Ga-FAPI points are more widely scattered, indicating moderate heterogeneity, yet consistently to the right of FDG’s values. Critically, the mean log(DOR) for FAPI is much higher than for FDG, implying far better diagnostic performance of FAPI PET/CT in gastric carcinoma supporting the meta-analytic conclusion that ^68^Ga-FAPI PET/CT has superior overall accuracy to ^18^F-FDG PET/CT in gastric cancer

 After adjustment, the pooled log[DOR] decreased slightly (from ~4.65 to ~4.43), but the overall conclusion that ^68^Ga-FAPI PET/CT has very high diagnostic accuracy remains unchanged. In other words, the sensitivity analysis indicates that the primary result is robust to potential bias, and any unpublished studies would slightly attenuate but not negate the strong diagnostic effect. The adjusted contour funnel plot and pooled estimate provide reassurance that publication bias, if present, has only a limited impact on the overall diagnostic odds ratio (Figure 13).

 As seen, the pooled sensitivity and specificity were significantly higher for ^68^Ga-FAPI PET/CT, which correlates with its ability to detect tumors independent of glucose metabolism. Furthermore, ^68^Ga-FAPI PET/CT demonstrated enhanced tumor delineation, particularly in cases where ^18^F-FDG PET/CT is known to be limited, such as mucinous and diffuse-type gastric cancers.

 This feature significantly reduces false-negative results and enhances the delineation of tumor margins, which is crucial for accurate staging and treatment planning. Additionally, ^68^Ga-FAPI PET/CT has demonstrated a higher diagnostic accuracy in detecting peritoneal carcinomatosis, a common and challenging metastatic pathway in gastric carcinoma, which ^18^F-FDG PET/CT often fails to detect effectively.

 From a clinical management perspective, the superior imaging characteristics of ^68^Ga-FAPI

PET/CT offer potential benefits in surgical decision making, radiation therapy planning, and monitoring treatment response. The ability to better visualize tumor burden can aid in refining therapeutic strategies and optimizing patient outcomes. Furthermore, it may play a role in the early identification of recurrence and residual disease, which is critical for timely intervention.

 A key statistical analysis performed in our study was DeLong’s test (Table 4) to compare the areas under the summary receiver operating characteristic (SROC) curves. The test yielded a highly significant p-value of 0.0013, confirming that the difference in diagnostic accuracy between ^68^Ga FAPI PET/CT (AUC=0.96; 95% C.I: 0.899 - 1.000) and ^18^F-FDG PET/CT (AUC=0.76; 95% C.I: 0.654 - 0.864) for primary gastric carcinoma is statistically significant. Even more significantly the AUC=0.97 and p-value ~2.6×10-5 for ^68^Ga-FAPI PET/CT were much higher for detection of omental metastasis (Table 4). This finding further strengthens the argument for adopting ^68^Ga-FAPI PET/CT as a superior imaging modality for gastric cancer detection, staging and recurrence detection.

**Table 4:** Showing PPV, NPV, and accuracy of ^68^Ga-FAPI and ^18^F-FDG PET /CT for detection of primary gastric carcinoma. I2, Cochran’s Q and τ are heterogeneity statistic (df = k–1 = 13). τ² is the estimated between-study variance; τ = √τ² is its square-root. All values based on a DerSimonian–Laird random-effects model with 0.5 continuity correction and inverse-variance weighting

Metric	Modality	Pooled Estimate (%)	95 % CI	I²	Q (df = 13)	*p*-value	τ²	τ
PPV	^68^Ga-FAPI PET/CT	98.7	96.8–99.4	12 %	14.8	0.30	0.002	0.045
	^18^F-FDG PET/CT	89.3	85.4–92.1	25 %	16.2	0.22	0.008	0.089
NPV	^68^Ga-FAPI PET/CT	80.1	71.2–86.5	69 %	44.5	< 0.001	0.035	0.187
	^18^F-FDG PET/CT	56.8	48.9–64.4	60 %	38.2	< 0.001	0.028	0.167
Accuracy	^68^Ga-FAPI PET/CT	94.3	91.6–96.2	30 %	19.5	0.10	0.005	0.071
	^18^F-FDG PET/CT	75.8	71.0–80.1	35 %	22.8	0.04	0.010	0.100

Clinically, ^68^Ga-FAPI PET/CT holds promise for refining TNM staging, particularly in identifying occult peritoneal metastases, thereby guiding surgical decisions and avoiding futile interventions (24). Its prognostic value, evidenced by the correlation between high FAPI uptake and shorter progression free survival (HR: 2.4), aligns with FAP’s role in promoting tumor invasiveness and immune-suppression (24, 25). This positions ^68^Ga-FAPI PET/CT as a potential biomarker for risk stratification and therapeutic monitoring, especially in FAP targeted therapy trials (27).

 Overall, the evidence strongly supports the clinical utility of ^68^Ga-FAPI PET/CT in gastric carcinoma imaging, highlighting its potential to replace or complement ^18^F-FDG PET/CT in routine oncologic imaging. However, despite all above described advantages, there are certain limitations of ^68^Ga-FAPI PET/CT, which must be acknowledged. First, while it exhibits high sensitivity, its specificity, though comparable to ^18^F-FDG PET/CT, may be influenced by the presence of non-malignant fibroblast activation, potentially leading to false positive results (27, 28). Additionally, the availability of ^68^Ga-FAPI tracers remains limited, and their production requires specialized infrastructure, which may not be readily accessible in all healthcare settings. Another drawback is the standardi-zation of imaging protocols and cut-off values for FAPI uptake are still required to improve reproducibility across different centers (29, 30).

 Therefore, further large scale, prospective multicenter studies are needed to confirm the diagnostic superiority of ^68^Ga-FAPI PET/CT across diverse patient populations. Further research should also explore its role in treatment monitoring and response assessment, particularly in comparison to conventional imaging techniques.

### Summary of main findings and clinical Implications

 The pooled results demonstrate that ^68^Ga-FAPI PET/CT exhibits high sensitivity and specificity, high lesion to background contrast and favorable diagnostic accuracy in detecting primary gastric tumors as well as metastatic lesions, particularly in cases where ^18^F-FDG PET/CT is known to be suboptimal, such as diffuse type and mucinous gastric cancers. Furthermore, studies that performed head to head comparisons consistently reported superior or complementary diagnostic value of ^68^Ga-FAPI over ^18^F-FDG PET/CT, especially for peritoneal and lymph node metastases. The superior imaging characteristics of ^68^Ga-FAPI PET/CT support its potential integration into clinical workflows for gastric cancer staging and restaging. Improved detection of occult metastases particularly in the peritoneum or lymph nodes could significantly influence treatment planning, including surgical decisions and systemic therapy eligibility. 

 Additionally, its performance in low FDG avid histologies suggests that ^68^Ga-FAPI PET/CT may reduce the number of false negatives in these challenging subtypes. This focused meta-analysis evaluating specific role of ^68^Ga-FAPI PET/CT in diagnosis and staging of patients with gastric cancer, provided a robust synthesis of its diagnostic performance as well as its comparative performance to ^18^F-FDG PET/CT and hence offering further clinical value.

### Strengths and Limitations

 The main strengths of this systemic review include a comprehensive search strategy, strict adherence to PRISMA guidelines, and rigorous risk of bias assessment using QUADAS-2 tool. The inclusion of available FDG-PET/CT data from some of these studies, for comparative analysis though not fully but somewhat strengthens the clinical relevance of our conclusions.

 A few limitations must be acknowledged for this meta-analysis. First, most included studies included and analyzed were single center and had small sample sizes, which may limit generalizability. Second, heterogeneity in study design, reference standards, and imaging protocols may have influenced the pooled estimates. Third, direct comparison with FDG PET/CT was done/ not available in all 14 studies included in the analysis. Fourth, bias risk, though modest was observed. However, despite the modest bias risk, the meta-analysis lead to acceptable evidence showing the superiority of ^68^Ga FAPI PET/CT over ^18^F-FDG PET/CT for detection, staging and restaging of gastric carcinoma.

## Conclusion

 In summary, this systematic review and meta-analysis demonstrates that ^68^Ga-FAPI PET/CT has high diagnostic accuracy for staging of gastric carcinoma and, in the available head to head data, tends to outperform ^18^F-FDG PET/CT for detection of primary tumors and several key categories of metastases, particularly omental and skeletal lesions. These findings support the role of ^68^Ga-FAPI PET/CT as a promising complementary imaging modality for staging and restaging gastric cancer, especially in patients with low FDG avidity or suspected peritoneal spread. Nevertheless, PET/CT does not replace endoscopy and histology for diagnosis, and current evidence is insufficient to claim superiority in treatment response evaluation. Robust, well designed prospective studies are needed before ^68^Ga-FAPI PET/CT can be recommended for routine use in clinical guidelines.
